# Applied Solutions to Balance Conservation Need With Practical Applications: A Case Study With Eagles Movement Models and Wind Energy Development

**DOI:** 10.1002/ece3.71344

**Published:** 2025-04-25

**Authors:** M. Murgatroyd, A. Amar

**Affiliations:** ^1^ HawkWatch International Salt Lake City Utah USA; ^2^ FitzPatrick Institute of African Ornithology University of Cape Town Cape Town South Africa; ^3^ The Endangered Wildlife Trust Johannesburg South Africa

**Keywords:** collision risk, green‐green dilemma, movement ecology, raptors, renewable energy, sustainable development

## Abstract

The wind energy industry presents a green‐green dilemma whereby it aims to reduce CO_2_ emissions and combat climate change, benefiting biodiversity, but its development also negatively impacts biodiversity. To reconcile this, the first action in the mitigation hierarchy is to avoid development in high‐risk areas for vulnerable species. For raptors, development is often restricted within a certain distance from nests, or more recently, by using predictive habitat use models to define site‐ and species‐specific areas of high collision risk. One such model has been used to predict areas of high collision risk where development should be avoided for Verreaux's Eagles ( *Aquila verreauxii* ) in South Africa, but industry use of this tool has declined (research‐implementation gap, RIG). Uncertainty over the model outputs is a likely cause of the RIG because the model results in variably sized exclusion areas for each development. To reduce this uncertainty and increase implementation of the model, we explore if limiting these predicted risk areas to protect the same amount of space or less, as a circular buffer around the nest, provides improved protection for the species. We found that by fixing the area of risk to be equal to the area of the current circular buffer recommendation, eagle protection, that is, the proportion of space used by eagles that is protected, was improved by around 6%–7% compared to circular buffers or by 2%–3% compared to previous threshold‐based classifications. This fixed‐area approach ensures that by applying the collision risk potential model there is no unexpected loss in developable area for wind energy developers. Our study demonstrates the importance of understanding and adapting tools that aim to promote sustainable development of renewable energy. Responding to stakeholder needs and balancing conservation with practical applications is critical, particularly in countries where policy enforcement is lacking.

## Graphical abstract

Wind energy development is known to negatively impact birds through collisions with turbines. Predictive space use models can be useful for identifying areas which should be designated for the conservation of vulnerable species. However, uncertainty over modelled outputs can create user wariness. We review risk classification methods to standardise outputs and improve industry use, and ultimately improve protection of eagles.

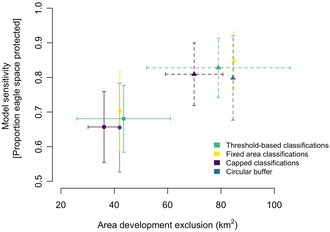

## 1 Introduction

A global transition to renewable energy is fundamental for climate change mitigation (York and Bell 2019). In South Africa, the Integrated Resource Plan (IRP) has set a target of increasing the renewable energy component of the power generation capacity from the current 11% to 40% by 2030. This would require the installation of around 17.7 GW of wind, equivalent to nearly six times the currently installed capacity (Department of Energy 2019).

While renewable energy is generally accepted as a desirable ‘green’ initiative to combat climate change, it can also have negative impacts on the environment (Voigt et al. 2019). For example, raptors are particularly prone to collisions with wind turbines, and this can result in population‐level declines, even for relatively common species (Duriez et al. 2022). Thus wind energy development and biodiversity conservation can present a green‐green dilemma, requiring a balance between renewable energy growth and wildlife protection (Voigt et al. 2019).

Strategies to mitigate the impacts of renewable energy developments are ranked within the mitigation hierarchy, with the first tier mitigation and most important action being the avoidance of development in areas where it could pose a threat to vulnerable species (Arnett and May 2016; Ekstrom et al. 2013). For this to occur, some spatial analysis of pre‐construction use, biodiversity value, or habitat suitability for vulnerable species at proposed development sites is required (Jones et al. 2022). More recently, applied predictive tools have been developed to identify areas which may be sensitive to wind energy developments; these include multi‐species sensitivity maps (Bright et al. 2008) and predictive habitat use or collision risk potential models (3D habitat use) (Cervantes et al. 2023; Mcleod et al. 2002; Murgatroyd et al. 2021; Reid et al. 2015; Scacco et al. 2022).

For raptors, predictive habitat use or collision risk potential models are generally species‐specific and use tracking data to quantify the relationships between flight and environmental variables in such a way that space use can be predicted across the landscape at proposed development sites (Mcleod et al. 2002; Murgatroyd et al. 2021; Reid et al. 2015; Scacco et al. 2022). A key advantage of such models in the wind energy industry is their ability to rapidly quantify risk to a species during the early stages of planning, allowing for the avoidance of turbine placement in locations that pose high risk to vulnerable species (Murgatroyd et al. 2021; Ralston‐Paton and Murgatroyd 2021; Tikkanen et al. 2018). While model‐based approaches will not completely replace the need for field surveys, for developers they are advantageous as they can save planning resources and time by (i) minimising large investments in planning developments at locations where the impact on biodiversity could lead to rejection of proposed wind energy facilities by permitting authorities, and (ii) reducing subsequent surveys and mitigation requirements for approved wind farms, both of which contribute to speeding up societies transitioning to renewable energy (Jones et al. 2022; Ralston‐Paton and Murgatroyd 2021; York and Bell 2019).

### 1.1 Verreaux's Eagles and Wind Energy

In South Africa, Verreaux's Eagles ( *Aquila verreauxii* ) are the most frequently listed raptor species of concern within wind energy Environmental Impact Assessments (EIAs), being resident at around 60% of proposed developments (Ralston‐Paton 2017). Verreaux's Eagles are territorial, non‐migratory, and use the same nest site repeatedly over many years. Concerns over the number of collision mortalities for this species have led to the publication of Birdlife South Africa's (BLSA) best‐practice guidelines for this species in relation to wind energy development (Ralston‐Paton 2017; Ralston‐Paton and Murgatroyd 2021). Although BLSA guidelines are generally accepted as minimum standards to be used by industry, they are not a legal policy requirement. Nevertheless, these guidelines provide a minimum recommendations for pre‐construction monitoring and mitigation, including areas where development should not be permitted around nest sites. For Verreaux's Eagles, this was initially given as 3 km radius circular buffers centred on nests, an area that was derived from early tracking studies on the species (Murgatroyd et al. 2016; Ralston‐Paton 2017). However, following multiple collision mortalities outside of this buffer distance, this recommendation was deemed insufficient to protect the species (Ralston‐Paton and Murgatroyd 2021; Ralston Paton et al. 2017).

In response to this environmental challenge, Murgatroyd et al. (2021) produced a predictive model, based on data from 15 GPS tracked Verreaux's Eagles, which can be applied to determine collision risk potential on a site‐specific basis. The model uses topographic variables and locations of the nests of resident and conspecific eagles to determine spatially explicit collision risk for the footprint area of any proposed wind energy facility with nest locations within 12 km. Implementing this model can provide better protection for eagles (where eagle protection is measured as model sensitivity, or the proportion of space used by eagles correctly classified by the model) than an equivalent‐sized circular buffer. On average, it can also make more land available for development than a circular buffer while achieving the same level of eagle protection, which should be an incentive for the use of this model in the wind energy industry (Murgatroyd et al. 2021).

In 2021, use of this collision risk potential model termed ‘VERA’ (Verreaux's Eagle Risk Assessment) was included in the best‐practice guidelines for wind energy development and Verreaux's Eagles (Ralston‐Paton and Murgatroyd 2021). Additionally, these guidelines provided an incentive to developers for applying VERA and avoiding turbines in all areas predicted to pose a collision risk to resident eagles, in the form of a reduced pre‐construction assessment period (1 year instead of 2 years) (Ralston‐Paton and Murgatroyd 2021). In the first year of use (2021), the model was applied to 15 proposed developments. Although it is difficult to know how many of these were subsequently submitted for government approval, in the same year there were a total of 12 EIA scoping and Basic Assessment (BAR) reports submitted (DFFE 2025), suggesting a very high initial use of the model. However, in 2022 and 2023, the model was only applied to two proposed developments each year despite 58 and 13 submissions respectively each year of EIA Scoping and BAR reports for wind energy developments (DFFE 2025). Although some of these projects may not have had Verreaux's Eagles present, from conversations with developers and avifaunal specialists, it appears that this new reluctance to use the model stems from an uncertainty about what the model will produce for an individual development, and specifically the amount of space that will be earmarked as high risk and excluded from development. Given the lack of government enforcement of the use of best‐practise guidelines, it is this uncertainty that we attempt to address here in order to resolve the current research‐implementation gap.

### 1.2 Threshold‐Based Model Classifications Create Wariness

The uncertainty for developers arises because, unlike circular buffers, the total size of the area where development is prohibited, or not recommended, can vary between nest locations. In the VERA model, Youden thresholds (i.e., cut off values used to maximise sensitivity and specificity of the model classifications) were calculated during the model building process, and these represent optimal cut‐off points for the categorisation of risk predictions (Murgatroyd et al. 2021). Two numeric thresholds are used, allowing risk predictions (i.e., probabilities) to be classified as ‘low’ (predictions less than the most precautionary or conservative threshold), ‘medium’ (predictions more than the precautionary threshold, but less than the high risk threshold), or ‘high’ (predictions greater than the high threshold). As such, the total area classified as high or medium risk for wind energy development by the VERA model is unbounded meaning that the area of risk is unknown and dependent on the site characteristics (i.e., the values of the variables that are included in the predictive model), in particular the slope and the distance to conspecific nests, which vary between eagle territories and wind farms. Although VERA risk predictions often result in a smaller area classified as medium or high risk (and thus development of wind farms not supported) compared to buffer circles, this is not always the case. It is this uncertainty around the potential area impacted for development prior to running the model that appears to have created a wariness amongst developers and resulted in a substantial drop in the use of the model to help guide eagle conservation and wind energy development.

This reluctance is understandable, because in some cases areas classified as high or medium collision risk can be significantly larger than areas delineated by circular buffers. This occurs commonly for isolated nests with no identified conspecific neighbours within 12 km. Although this may well reflect increased ranging behaviour of isolated eagle pairs (i.e., in the absence of neighbouring pairs an individual may range further from its nest), the size of the areas where wind energy development is not supported due to collision risk potential for eagles can be unfeasibly large for wind energy developers. Additionally, such large areas may also be artefacts due to avifaunal surveyors failing to find other conspecific nests within 12 km of the target nest.

The observed shift back to using traditional circular buffers to define high risk areas is a bad outcome for the species' conservation, as the VERA model has been shown to provide better protection for eagles (Murgatroyd et al. 2021). Thus, in this study, we explore scenarios that might offer better overall protection for eagles by excluding development from more of the core areas used by eagles compared to circular buffers, while also producing realistic recommendations to guide wind energy development in a more sustainable way.

## 2 Methods

### 2.1 Testing a Solution

**FIGURE 1:**
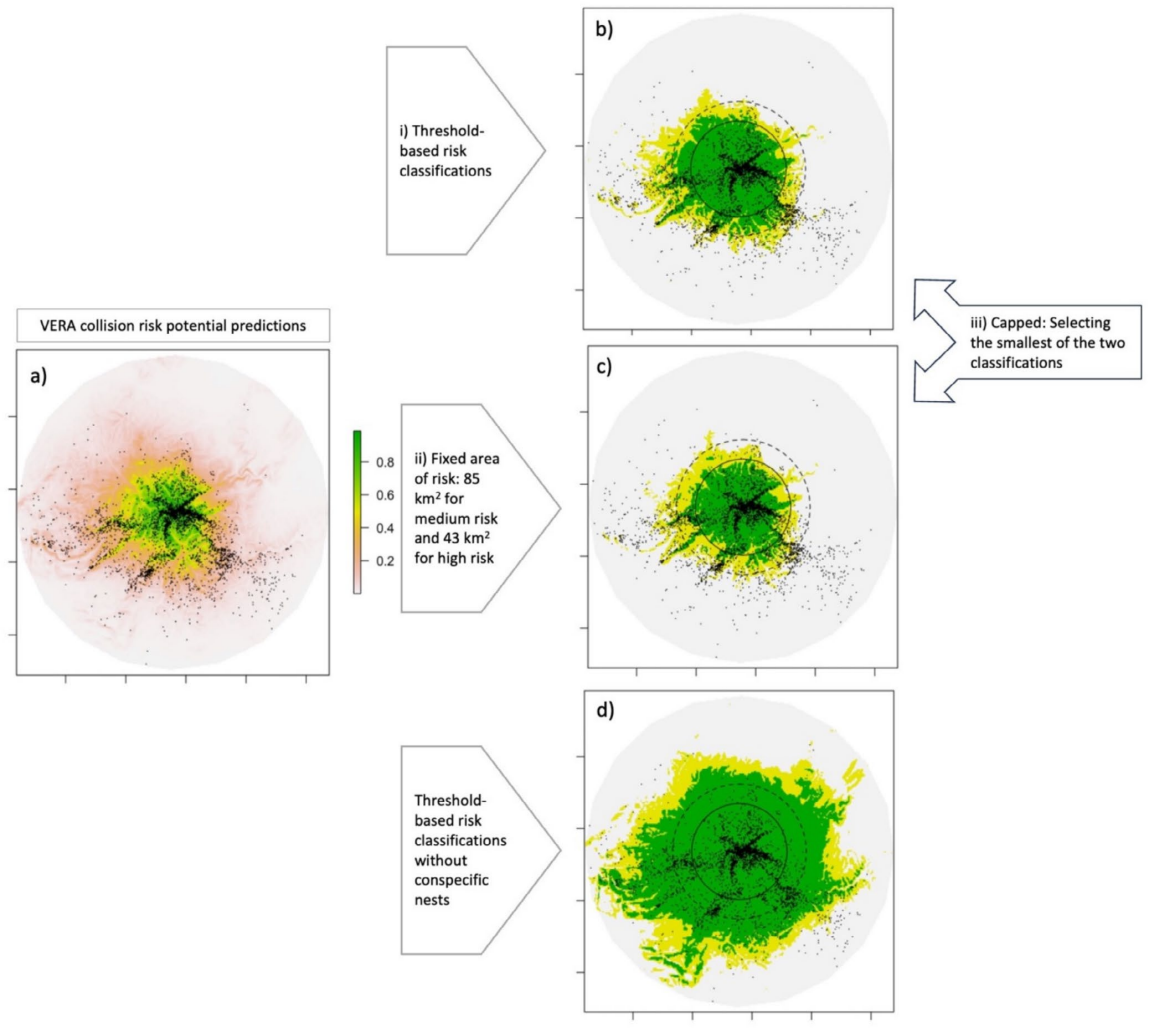
Illustration for one test eagle showing (a) the continuous predictions from the standard VERA model and tracking data collected over 1 year, and the size and shape of the categorical medium and high risk areas (where (b–d) high risk areas shown in green and the medium risk areas in yellow), depending on which classification method is used (i–iii): (b) threshold‐based classifications (baseline for current model performance), (c) area fixed to equal the buffer areas where high risk is 43 km^2^ (equivalent to a 3.7 km buffer, solid circle), and medium risk is 85 km^2^ (equivalent to a 5.2 km buffer, dashed circle) (to eliminate developer wariness caused by variable results), or (iii) capped risk classifications (to explore the impact on eagles if benefits to developers are maximised). Lastly, (d) shows threshold‐based classifications without conspecific nests (to confirm the extent to which the absence of conspecific nests can inflate predicted risk areas).

Using the same data and model as Murgatroyd et al. (2021), we examine the classification of the predictive outputs of the model and explore the impacts for developers and eagles on defining the risk areas using alternative methods (Figure 1). We test three variations for classifying the medium and high risk areas from the VERA model predictions and compare these to circular buffers and scenarios without conspecific nests. Each scenario is cross‐validated using data from seven test eagles (the same test eagles previously used to cross‐validate the VERA model, Murgatroyd et al. 2021) assessing the benefits to eagles (i.e., model sensitivity, the proportion of eagle space which the model protects) and the benefits to developers (i.e., minimising the area which development would be excluded from). The tests were:

1. The original threshold‐based classification of the VERA model, using the Youden thresholds to define risk classes (used as a baseline for current model performance) (Figure 1b).
2. A ‘fixed’ area classification of the VERA predictions, where the high and medium risk areas are equal to the current circular buffer recommendations (i.e., high risk: 43 km^2^ equivalent to a 3.7 km radius circular buffer and medium risk: 85 km^2^ equivalent to a 5.2 km radius circular buffer) (to eliminate developer wariness caused by variable results) (Figure 1c).
3. A ‘capped’ VERA classification, where the high and medium risk areas are not allowed to exceed the equivalent sized buffers (i.e., using whichever output had the smallest area from 1 and 2) (to explore the impact on eagles if benefits to developers are maximised) (Figure 1b,c).
4. The original threshold‐based classification of the VERA model, but the variable ‘distance to conspecific nest’ set to the maximum value. This model is equivalent to not having any conspecific nests in the surrounding area (to confirm the extent to which the absence of conspecific nests can inflate predicted risk areas) (Figure 1d).
5. Circular buffer exclusions around nests (to explore eagle protection based on fixed buffers) (Figure 1).

## 3 Results

We found that in the absence of conspecific nests, the VERA model predicts larger areas of risk, which were on average 148% (for the medium risk area) to 205% (for the high risk area) greater than the area of equivalent circular buffers (Figure S1). This is in line with our expectation that when using the Youden thresholds, the lack of conspecific nests surrounding a development can lead to larger areas of habitat classified as being risky for this species. This reflects eagle ranging behaviour (i.e., eagles are more likely to use areas where there are no conspecifics (Murgatroyd et al. 2021)), but such large areas can be problematic for development.

**FIGURE 2:**
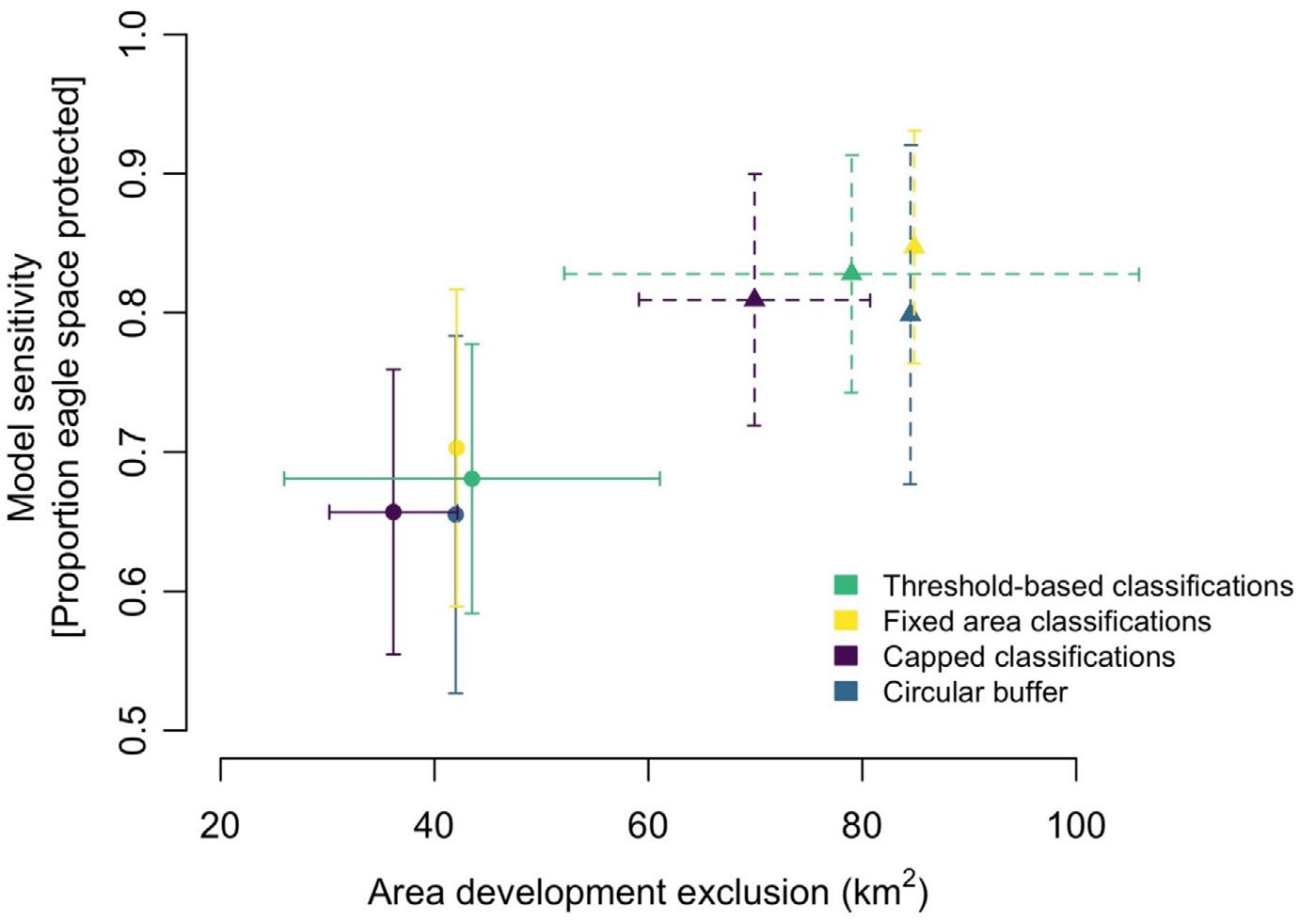
Model performance comparisons using the original threshold‐based classification of the VERA model, a ‘fixed’ area classification of the VERA predictions (where the high and medium risk areas are equal to the current circular buffer recommendations), a ‘capped’ VERA classification (where the high and medium risk areas are not allowed to exceed the equivalent sized buffers), and the standard circular buffers. Solid lines and dots show comparisons at the high risk threshold or equivalent 3.7 km buffer, dashed lines and triangles show comparisons at the medium risk threshold or equivalent 5.2 km buffer. Both show the mean and standard deviation of seven individual test eagles.

The original threshold‐based classification of the VERA predictions resulted in 4% higher model sensitivity (i.e., protected 4% more eagle space) compared to a circular buffer at both the medium and high risk thresholds. At the medium risk threshold this level of increased protection was achieved with, on average, 7% less space where development should be avoided compared to a 5.2 km circular buffer. However, at the high risk threshold, the model earmarked 4% more area from which development would be excluded as compared with the 3.7 km high risk buffer circle (Figure 2). Examining these results at an individual test eagle level, we found that the threshold‐based classifications resulted in medium risk areas which were larger than circular buffers < 30% of the time (2/7 cases), and in those cases risk areas were 23%–53% larger than circular buffers. However, more often (5/7 cases) the medium risk areas were 18%–30% smaller than circular buffers. Similarly, for the predicted high risk areas, the threshold‐based results classified larger areas as risky for two of the seven test eagles, and these areas were 46%–77% larger than the equivalent buffer circle. For the other five eagles with smaller areas, these predicted high risk areas were 2%–33% smaller than a circular buffer (Figure S1).

By fixing the area of the VERA predictions to equal the size of the circular buffer, average eagle protection was improved by 6 (medium risk) to 7 (high risk) % compared to the circular buffers (Figure 2 yellow vs. blue), and by 2 (medium risk) to 3 (high risk) % compared to the threshold‐based classifications (Figure 2 yellow vs. green).

By capping the area of the VERA predictions to never exceed the circular buffer predictions (but could be smaller), average eagle protection was only slightly more than the circular buffer (by 1.3% for medium risk and 0.3% for high risk) (Figure 2 purple vs. blue), and was less than the threshold‐based classifications (by 2.3% for medium risk and 3. 5% for high risk) (Figure 2 purple vs. green).

The mean area was similar between threshold‐based model classifications, fixed area, and circular buffers. However, the latter two methods removed individual variation in area (Figure 2 yellow and blue), while the threshold‐based classifications resulted in the largest variations in risk area (Figure 2 green error bars). The capped method resulted in the smallest risk areas overall (Figure 2 purple). The mean eagle protection was highest using the fixed area method and lowest using circular buffers (Figure 2).

## 4 Discussion

To benefit the overall conservation of Verreaux's eagles, a threatened raptor vulnerable to wind turbine collisions, we have re‐assessed the way the VERA model classifies collision risk potential. By standardising the size of the area that is flagged as high risk for wind energy development for this species, we hope that the available collision risk potential model will be applied more readily to avoid development in core areas used by resident eagles. We base our recommendation for a ‘fixed area’ classification of collision risk potential on the fact that it offers improved overall eagle protection, while also not costing the developer any more space than traditional circular buffers, by focusing protection on the areas more likely to be used by eagles. Indeed we found that this method offers better eagle protection on average, even compared to the original threshold‐based applications.

Avoiding harmful development in locations used by vulnerable species is the most fundamental step in the mitigation hierarchy (Arnett and May 2016; Ekstrom et al. 2013). This research has further confirmed that predictive models can be more useful than simple buffer methods for determining important areas for conservation, particularly related to the development of wind energy (Bright et al. 2008; Reid et al. 2015; Santos et al. 2013). Despite the availability of research tools to identify areas where development should be avoided, the lack of implementation of such tools can result in wind farms emerging without assessing avifaunal impacts to an appropriate level and potentially negatively impacting biodiversity. Ensuring the best available information and decision‐making tools are used for assessing the impacts of development is a widely recognised challenge in sustainable development and conservation (Addison et al. 2013; Gontier et al. 2006).

In order to increase buy‐in of predictive tools, like VERA, and thus close the gap between the knowledge generated by researchers and the information being used to inform policy and practice (research‐implementation gap) there is a need to respond and adapt these tools to stakeholder requirements (Addison et al. 2013; Dubois et al. 2020; Nkomo et al. 2024). Research needs to ensure that the models are beneficial to biodiversity conservation but also appropriate and practical for the end users and decision makers (Schuwirth et al. 2019). Ensuring that they meet both objectives can benefit both conservation aims (e.g., protection for eagles) and development aims (e.g., minimising the development area excluded) to improve overall green–green objectives.

To ensure best practise implementation across all developments, we recommend that the published guidelines are used as mandatory policies by the authorising bodies (Arnett and May 2016). However, where national legislation to quantify impacts of wind farm development on birds exists, there can still be inconsistency in methods of data collection or modelling, and in some cases current guidance does not reflect the most reliable methods available (Largey et al. 2021; Madders and Whitfield 2006). The decision‐making process in energy development planning is complex and needs to take into account multiple biodiversity and socio‐economic factors, often choosing trade‐offs between development and conservation (Kareksela et al. 2018; Weber et al. 2023). However, because large raptors are a frequent concern for avifaunal assessments at wind energy sites, it is important to have clear guidance to avoid potential population declines (Cervantes et al. 2022). In countries where there is a lack of clear guidance or enforcement of best practice guidelines in the EIA process, responding to stakeholder needs and balancing conservation with practical applications is critical. It is in everyone's interest, developers and conservationists alike, to ensure that any obstacles to using quantitative tools for risk assessments are identified and overcome so that research is appropriately implemented within the EIA process.

## Author Contributions

**M. Murgatroyd:** conceptualization (equal), data curation (lead), formal analysis (lead), methodology (equal), writing – original draft (lead), writing – review and editing (equal). **A. Amar:** conceptualization (equal), formal analysis (supporting), methodology (supporting), writing – original draft (supporting), writing – review and editing (equal).

## Conflicts of Interest

The authors declare no conflicts of interest.

## Supporting information


Figure S1.

## Data Availability

Data available via the Dryad Digital Repository https://doi.org/10.5061/dryad.b5mkkwhc1 (Murgatroyd et al. 2021).

## References

[ece371344-bib-0001] Addison, P. F. E. , L. Rumpff , S. S. Bau , et al. 2013. “Practical Solutions for Making Models Indispensable in Conservation Decision‐Making.” Diversity and Distributions 19, no. 5–6: 490–502. 10.1111/ddi.12054.

[ece371344-bib-0002] Arnett, E. B. , and R. F. May . 2016. “Mitigating Wind Energy Impacts on Wildlife: Approaches for Multiple Taxa.” Human‐Wildlife Interactions 10, no. 1: 28–41.

[ece371344-bib-0003] Bright, J. , R. Langston , R. Bullman , R. Evans , S. Gardner , and J. Pearce‐Higgins . 2008. “Map of Bird Sensitivities to Wind Farms in Scotland: A Tool to Aid Planning and Conservation.” Biological Conservation 141, no. 9: 2342–2356. 10.1016/j.biocon.2008.06.029.

[ece371344-bib-0004] Cervantes, F. , M. Martins , and R. E. Simmons . 2022. “Population Viability Assessment of an Endangered Raptor Using Detection/Non‐Detection Data Reveals Susceptibility to Anthropogenic Impacts.” Royal Society Open Science 9, no. 2: 220043. 10.1098/rsos.220043. 35223069 PMC8864359

[ece371344-bib-0005] Cervantes, F. , M. Murgatroyd , D. Allan , et al. 2023. “A Utilization Distribution for the Global Population of Cape Vultures *Gyps coprotheres* to Guide Wind Energy Development.” Ecological Applications 33: e2809. 36691259 10.1002/eap.2809

[ece371344-bib-0030] Department of Energy . 2019. “Integrated Resource Plan 2019.” Department of Energy, Pretoria, Republic of South Africa. https://www.dmre.gov.za/Portals/0/Energy_Website/IRP/2019/IRP‐2019.pdf.

[ece371344-bib-0006] DFFE . 2025. “Renewable Energy EIA Application Database for SA.” https://egis.environment.gov.za/renewable_energy.

[ece371344-bib-0007] Dubois, N. S. , A. Gomez , S. Carlson , and D. Russell . 2020. “Bridging the Research‐Implementation Gap Requires Engagement From Practitioners.” Conservation Science and Practice 2, no. 1: 1–12. 10.1111/csp2.134.

[ece371344-bib-0008] Duriez, O. , P. Pilard , N. Saulnier , P. Boudarel , and A. Besnard . 2022. “Windfarm Collisions in Medium‐Sized Raptors: Even Increasing Populations Can Suffer Strong Demographic Impacts.” Animal Conservation 26, no. 2: 1–12. 10.1111/acv.12818.

[ece371344-bib-0009] Ekstrom, J. , L. Bennun , and R. Mitchell . 2013. “A Cross‐Sector Guide for Implementing the Mitigation Hierarchy.” The Biodiversity Consultancy Ltd. http://www.riotinto.com/sustainabledevelopment2013/environment/biodiversity.html.

[ece371344-bib-0010] Gontier, M. , B. Balfors , and U. Mörtberg . 2006. “Biodiversity in Environmental Assessment‐Current Practice and Tools for Prediction.” Environmental Impact Assessment Review 26, no. 3: 268–286. 10.1016/j.eiar.2005.09.001.

[ece371344-bib-0011] Jones, K. R. , A. von Hase , H. M. Costa , et al. 2022. “Spatial Analysis to Inform the Mitigation Hierarchy.” Conservation Science and Practice 4, no. 6: 1–17. 10.1111/csp2.12686.

[ece371344-bib-0012] Kareksela, S. , A. Moilanen , O. Ristaniemi , R. Välivaara , and J. S. Kotiaho . 2018. “Exposing Ecological and Economic Costs of the Research‐Implementation Gap and Compromises in Decision Making.” Conservation Biology 32, no. 1: 9–17. 10.1111/cobi.13054. 29139572

[ece371344-bib-0013] Largey, N. , A. S. C. P. Cook , C. B. Thaxter , et al. 2021. “Methods to Quantify Avian Airspace Use in Relation to Wind Energy Development.” Ibis 163, no. 3: 747–764. 10.1111/ibi.12913.

[ece371344-bib-0014] Madders, M. , and D. P. Whitfield . 2006. “Upland Raptors and the Assessment of Wind Farm Impacts.” Ibis 148, no. s1: 43–56. 10.1111/j.1474-919X.2006.00506.x.

[ece371344-bib-0015] Mcleod, D. R. A. , D. P. Whitfield , A. H. Fielding , P. F. Haworth , and M. J. Mcgrady . 2002. “Predicting Home Range Use by Golden Eagles *Aquila chrysaetos* in Western Scotland.” Avian Science 2: 1–17.

[ece371344-bib-0016] Murgatroyd, M. , W. Bouten , and A. Amar . 2021. “A Predictive Model for Improving Placement of Wind Turbines to Minimise Collision Risk Potential for a Large Soaring Raptor.” Journal of Applied Ecology 58, no. 4: 857–868. 10.1111/1365-2664.13799.

[ece371344-bib-0017] Murgatroyd, M. , L. G. Underhill , W. Bouten , and A. Amar . 2016. “Ranging Behaviour of Verreaux's Eagles During the Pre‐Breeding Period Determined Through the Use of High Temporal Resolution Tracking.” PLoS One 11, no. 10: e0163378. 10.1371/journal.pone.0163378. 27723832 PMC5056708

[ece371344-bib-0018] Nkomo, M. N. , M. Murgatroyd , S. Ralston‐Paton , and A. Amar . 2024. “Using Stakeholder Knowledge to Co‐Produce Research Priorities for a Raptor Species Vulnerable to Impacts of Wind Energy Facilities.” Ostrich 95, no. 3: 200–212. 10.2989/00306525.2024.2387723.

[ece371344-bib-0019] Ralston Paton, S. , J. Smallie , A. Pearson , and R. Ramalho . 2017. Wind Energy's Impacts on Birds in South Africa: A Preliminary Review of the Results of Operational Monitoring at the First Wind Farms of the Renewable Energy Independent Power Producer Procurement Programme in South Africa. BirdLife South Africa.

[ece371344-bib-0020] Ralston‐Paton, S. 2017. Verreauxs' Eagle and Wind Farms: Guidelines for Impact Assessment, Monitoring, and Mitigation. BirdLife South Africa.

[ece371344-bib-0021] Ralston‐Paton, S. , and M. Murgatroyd . 2021. Verreaux's Eagle and Wind Farms: Guidelines for Impact Assessment, Monitoring and Mitigation. BirdLife South Africa.

[ece371344-bib-0022] Reid, T. , S. Krüger , D. P. Whitfield , and A. Amar . 2015. “Using Spatial Analyses of Bearded Vulture Movements in Southern Africa to Inform Wind Turbine Placement.” Journal of Applied Ecology 52, no. 4: 881–892. 10.1111/1365-2664.12468.

[ece371344-bib-0023] Santos, H. , L. Rodrigues , G. Jones , and H. Rebelo . 2013. “Using Species Distribution Modelling to Predict Bat Fatality Risk at Wind Farms.” Biological Conservation 157: 178–186. 10.1016/j.biocon.2012.06.017.

[ece371344-bib-0024] Scacco, M. , E. Arrondo , J. A. Donazar , et al. 2022. “The Species‐Specificity of Energy Landscapes for Soaring Birds, and Its Consequences for Transferring Suitability Models Across Species.” Landscape Ecology 38, no. 1: 239–252. 10.1007/s10980-022-01551-4.

[ece371344-bib-0025] Schuwirth, N. , F. Borgwardt , S. Domisch , et al. 2019. “How to Make Ecological Models Useful for Environmental Management.” Ecological Modelling 411, no. May: 108784. 10.1016/j.ecolmodel.2019.108784.

[ece371344-bib-0026] Tikkanen, H. , S. Rytkönen , O. P. Karlin , et al. 2018. “Modelling Golden Eagle Habitat Selection and Flight Activity in Their Home Ranges for Safer Wind Farm Planning.” Environmental Impact Assessment Review 71, no. September 2017: 120–131. 10.1016/j.eiar.2018.04.006.

[ece371344-bib-0027] Voigt, C. C. , T. M. Straka , and M. Fritze . 2019. “Producing Wind Energy at the Cost of Biodiversity: A Stakeholder View on a Green‐Green Dilemma.” Journal of Renewable and Sustainable Energy 11, no. 6: 063303. 10.1063/1.5118784.

[ece371344-bib-0028] Weber, J. , T. Steinkamp , and M. Reichenbach . 2023. “Competing for Space? A Multi‐Criteria Scenario Framework Intended to Model the Energy–Biodiversity–Land Nexus for Regional Renewable Energy Planning Based on a German Case Study.” Energy, Sustainability and Society 13, no. 1: 1–33. 10.1186/s13705-023-00402-7.

[ece371344-bib-0029] York, R. , and S. E. Bell . 2019. “Energy Transitions or Additions?: Why a Transition From Fossil Fuels Requires More Than the Growth of Renewable Energy.” Energy Research & Social Science 51: 40–43. 10.1016/j.erss.2019.01.008.

