# Supplementary material for: Applied Solutions to Balance Conservation Need With Practical Applications: A Case Study With Eagles Movement Models and Wind Energy Development
Source: Ecol Evol. 2025 Apr 25;15(4):e71344. doi: 10.1002/ece3.71344 (PMC12022779; doi:10.1002/ece3.71344)
Supplement: Supplementary file 1 — Figure S1. [file ECE3-15-e71344-s001.docx]

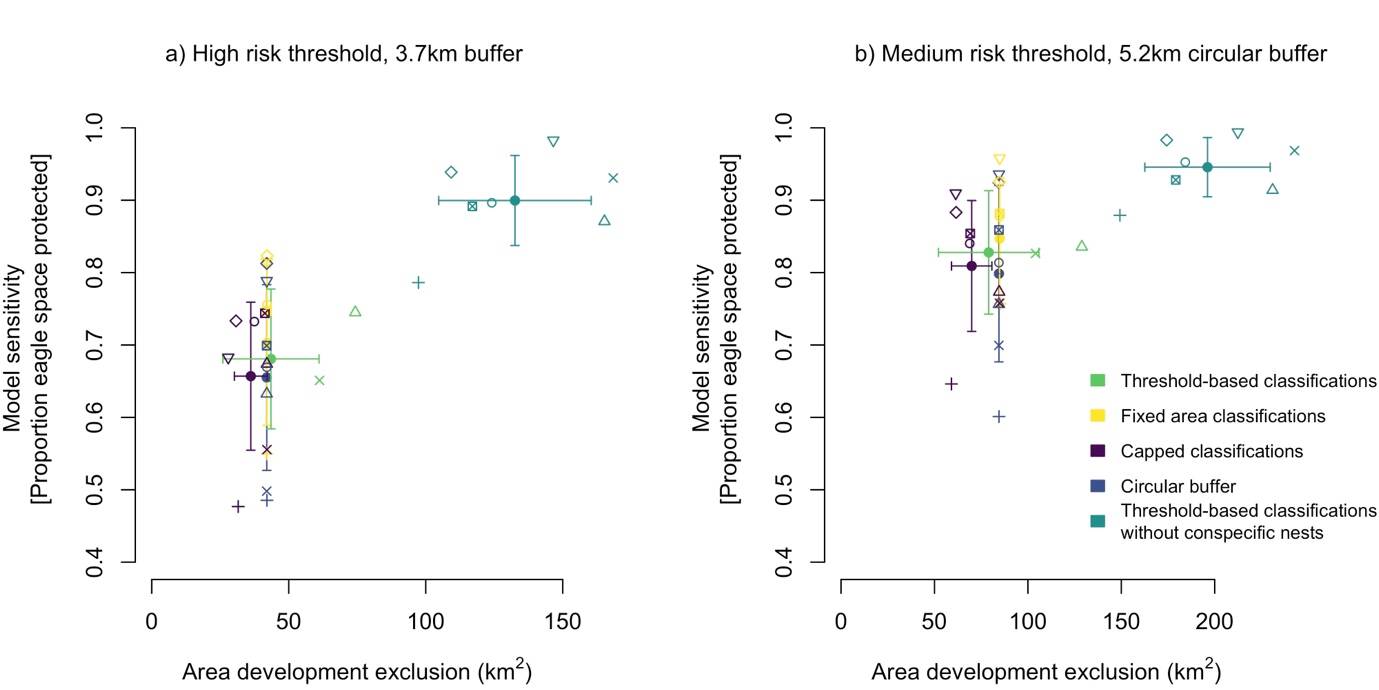


Figure S1. Model performance comparisons using the original unbounded VERA model, a ‘fixed’ area classification of the VERA predictions (where the high and medium risk areas are equal to the current circular buffer recommendations), a ‘capped’ VERA classification (where the high and medium risk areas are not allowed to exceed the equivalent sized buffers), the original unbounded VERA model with the variable for distance to conspecific nest set to the maximum, and the standard circular buffers. a) shows comparisons at the medium risk threshold, equivalent to a 5.2km buffer, b) shows comparisons at the high risk threshold, equivalent to a 3.7km buffer. Dots and solid lines show the mean and standard deviation of seven individual test eagles which are shown by unique symbols.
